# Neuromyelitis optica in a Ugandan woman: a case report

**DOI:** 10.1186/1752-1947-7-177

**Published:** 2013-07-05

**Authors:** Mark Kaddumukasa, Abdu Musubire, Martin Kaddumukasa, Steven Matovu, Elly Katabira

**Affiliations:** 1 Neurology Unit, Department of Medicine, College of Health Sciences, Makerere University, P. Box 7072, Kampala, Uganda

**Keywords:** Uganda, Neuromyelitis optica, Devic’s syndrome

## Abstract

**Introduction:**

Few cases of neuromyelitis optica have been reported in Africa. This is the first case report of neuromyelitis optica in Uganda. It highlights the need to have a high index of suspicion to promptly identify and appropriately treat these patients.

**Case presentation:**

We present the case of a 24-year-old woman of Bantu origin who presented initially with bilateral loss of vision and weakness of the lower limbs in 2010 that resolved completely after a few days. Eight months later, she presented with bilateral lower limb weakness and urinary incontinence that improved completely following steroid use. This was followed four months later with an episode of quadriparesis that was treated with steroids and azathioprine with some improvement currently using a walking aide.

**Conclusions:**

The patient described here represents a phenotypic expression of a recurrent (multiphasic), steroid-sensitive, inflammatory demyelinating disorder of the central nervous system occurring in a black Ugandan woman. This case highlights the occurrence of Devic’s disease within our setting and the need to properly diagnose this condition even in a resource-limited setting to prevent disability.

## Introduction

Neuromyelitis optica (Devic’s syndrome) is defined as a devastating myelitis with the following: an acute unilateral or bilateral optic neuropathy, no clinical involvement beyond the spinal cord or optic nerves, and a monophasic or, rarely, a multiphasic illness [1-3]. The discovery of neuromyelitis optica (NMO) immunoglobulin G (IgG), directed against aquaporin-4 (AQP4), has dramatically changed the clinical definition of NMO and is important in the diagnostic criteria of this disease [4,5]. However, in resource-limited centers this may be a limitation in meeting the formal diagnostic criteria pathway due to laboratory diagnostic setbacks.

No cases have been documented in Uganda and this is the first case reported in Uganda or surrounding equatorial regions in Sub-Saharan Africa. The low anticipation of such diseases in our setting, coupled with diagnostic challenges, often leads to misdiagnosis and worse outcomes in patients that could have benefited from readily available treatment. We believe the insights offered here may be useful to many other resource-limited settings.

## Case presentation

A 24-year-old African woman of Bantu origin presented to us in 2010 with sudden bilateral loss of vision and progressive weakness in both her lower limbs for one day. She had been previously well with no known chronic medical conditions, like hypertension or diabetes mellitus, and had no preceding vaccinations or viral infections noted. She has lived all her life in Uganda. Her brain computed tomography (CT) scan was normal and her blood test results were negative for polycythemia, thrombocytosis, and diabetes mellitus. Her systemic clinical examination, including the neurological assessment at the time of her initial medical presentation, was normal. She was treated as if she were a patient suffering a transient ischemic attack, with low-dose aspirin (ASA), which she later decided to stop over the course of time.

Eight months later, she presented to us with inability to walk and urinary incontinence for three days. This was preceded by parethesias in the lower limbs followed by unsteady gait and subsequently, an inability to use both her lower limbs and urinary incontinence. She reported no history of recent vaccinations, ingestion of tinned meats or beef and no recent sore throat. Her vision this time was normal with no complaints of double vision or visual field defects. Her upper limbs were normal. She was nulliparus and reported normal menses. She reported no history of hypertension or diabetes, and was not receiving any regular medications.

Clinically, she had muscle power grade 2 on the Medical Research Council (MRC) scale bilaterally, with spasticity and brisk reflexes of the knees and ankles bilaterally, with bilateral upgoing plantars. She had a symmetrical sensory deficit below T10 to fine touch and pressure. Her vision was normal with no visual field defects or double images and no optic atrophy or optic neuritis was detected on fundoscopy. She had urinary retention and a urinary catheter was placed *in situ*. Her series of blood test requests returned negative results for human immunodeficiency virus (HIV)1/2 testing and the treponema pallidum hemagglutination assay (TPHA)/Venereal Disease Research Laboratory test (VDRL) test for syphilis. Her peripheral blood film report revealed no malarial parasites or other hemoparasites. The full blood counts showed the following: the white blood cell count (WBC) was 10.2 × 10^3^/L, absolute neutrophils 7.1 × 10^3^/L, lymphocytes 1.3 × 10^3^, hemoglobin 11.6g/dL and platelets 252 × 10^3^. She had elevated acute-phase reactants with an erythrocyte sedimentation rate (ESR) of 25mm/hr (Westergren method) and C-reactive protein (CRP) of 17mg/L but her antinuclear antibodies test was negative. A lumbar puncture performed showed the cerebrospinal fluid (CSF) was clear and colorless, with a normal blood glucose level. However, the CSF protein content was elevated at 60mg/dL, while the WBC count in the CSF was less than 5 cells/uL. Indian ink staining on her CSF for *Cryptococcus* was negative and the Gram stain revealed no organisms. The Ziehl-Neelsen stain for tuberculosis revealed no acid-fast bacilli. The VDRL on the CSF was nonreactive and cytology revealed no malignant cells. She had no clinical evidence of sarcoidosis, vasculitis, systemic lupus erythematosus (SLE) or Sjogren’s syndrome. A brain and spinal magnetic resonance imaging (MRI) scan was requested for further evaluation.

**Figure 1:**
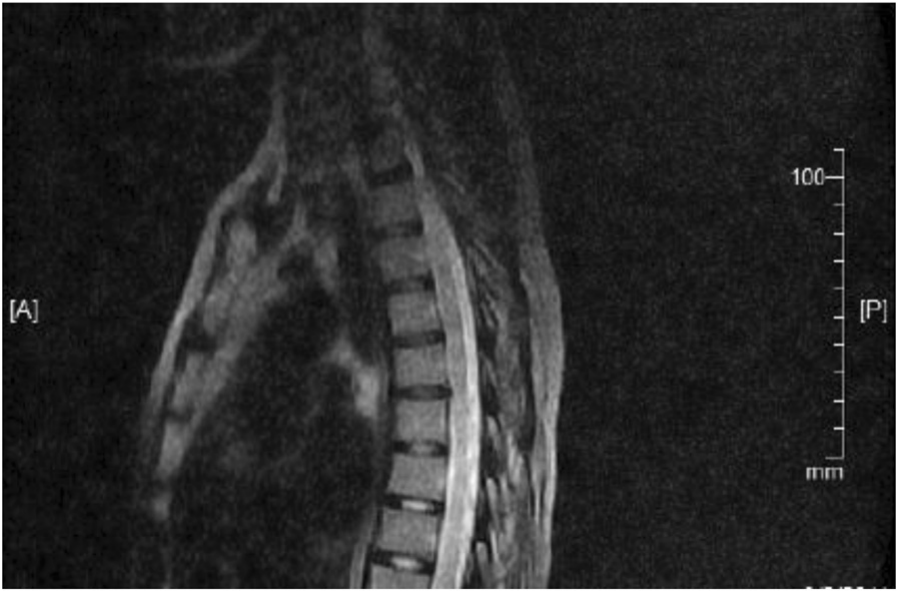
**Magnetic resonance imaging studies.** Sagittal T2-weighted magnetic resonance imaging showed hyperintense ill-defined lesions from T4 – T8.

Her MRI of the cervicothoracic spine revealed large multiple ill-defined hyperintense lesions involving the cervical and thoracic spinal cord up to the T4 to T8 vertebral levels on T2-weighted images (Figure 1). These appeared hypointense on T1-weighted images, probably representing myelitis or demyelination. No cord compressive lesions were found on her spinal MRI and her brain MRI was normal. A diagnosis of neuromyelitis optica was made based on the diagnostic criteria excluding the antibody assays [6].

She was started on pulse methyprednisolone 1g for five days and had complete resolution of her symptoms. She was discharged from hospital on tapering oral steroids but the patient stopped the low-dose oral steroids after two months of treatment. Due to lack of prior experience of the disease, she was not started on long-term steroid-sparing immunosuppressants like azathioprine or mycophenolate mofetil, which are readily available within the country though costly.

In February 2011, that is four months later, she returned to our care with sudden loss of sensation and numbness in the upper limbs and weakness in the lower limbs. Her power grading using the MRC scale was four minus in the upper limbs and two in the lower limbs respectively. We were unable to repeat the MRI scan due to limited resources and therefore unable to evaluate the presence and extent of any new lesions. The patient then received pulse therapy of methyprednisolone 1g once a day for five days, there was improvement in her lower limbs, and her power increased to four minus after about two weeks’ admission. She was able to walk with support and was started on daily tapered prednisolone and azathioprine starting at 2mg/kg/day, divided into two daily doses. She was not tested for the thiopurine S-methyltransferase (TPMT) mutation. Individuals who have this mutation can develop significant bone marrow toxicity with azathioprine; however, she gets a routine monthly blood check especially for the WBC counts. She was recommended to physiotherapy for muscle-strengthening exercises and bladder training. She has remained fairly stable, able to walk with the aid of a walking stick and is still under our follow-up care. She is currently maintained on azathioprine 100mg daily and the oral steroids have been gradually tapered off. We are continuing to monitor her WBC counts every month for leucopenia and liver function associated with azathioprine toxicity. She has remained stable and has not suffered any more relapses since then.

We are unable to perform the AQP4 antibodies test in our setting.

## Discussion

This case shows the complexities of diagnosing neuromyelitis optica in a resource-limited setting, especially in geographic areas initially thought not to have a burden of this disease. It is reported to be rare in our setting, though cases have been documented elsewhere [7-9]. And hence there is a need to adequately prepare for similar treatment challenges.

Therefore, a high index of suspicion is required to make this diagnosis early and illustrate the need for early and prompt diagnosis to delay unfavorable outcomes [1,3].

Meeting the 2006 revised diagnostic criteria of NMO [6] in our setting may still be difficult, especially with the serological assays. Clinicians need to rely on the other criterion and clinical judgments and initiate treatments so as to delay the consequences of the disease.

Our case highlights the fact that lack of the laboratory diagnostic criteria, such as detection of oligiclonal bands and of AQP4 antibodies, does not preclude the diagnosis and correct treatment approach in cases like the one described.

## Conclusions

Our case report is further strong evidence that neuromyelitis optica occurs in Uganda and Africa as a whole and requires a high index of suspicion and improvement in health-care provision to NMO patients in our setting. Proper training of medical personnel and students to promptly make a diagnosis and refer such cases for specialized care is required. Our patient had three episodes over a span of two years and suffered delays in instituting treatment due to lack of awareness as well as in planning long-term care for the patient. The availability of easier and faster assays would help in the decision-making of patient care. The limitations of determining oligoclonal bands and NMO-IgG antibodies in a resource-limited setting should not delay the process of making a diagnosis of NMO in tropical Africa.

## Consent

Written informed consent was obtained from the patient for publication of this case report and accompanying images. A copy of the written consent is available for review by the Editor-in-Chief of this journal.

## Abbreviations

AQP4: Aquaporin-4; CRP: C-reactive protein; CT: Computed tomography; ESR: Erythrocyte sedimentation rate; HIV: Human immunodeficiency virus; IgG: Immunoglobulin G; MRI: Magnetic resonance imaging; NMO: Neuromyelitis optica; SLE: Systemic lupus erythematosus; WBC: White blood cell.

## Competing interests

The authors declare that they have no competing interests.

## Authors’ contributions

All authors stated above made substantive intellectual contributions to the published case report. MK treated the patient and wrote the article. AM treated the patient and helped to draft the manuscript. SM participated in the treatment of the patient, helped to draft the manuscript and helped in making the diagnosis of the patient. MK helped to draft the manuscript. All authors read and approved the final manuscript.
